# Comparison of the lower limit of normal to the fixed ratio method for the diagnosis of airflow obstruction at high altitudes: a large cross-sectional survey of subjects living between 3000–4700 m above sea level

**DOI:** 10.1186/s40001-023-01151-1

**Published:** 2023-06-12

**Authors:** Yilin Huang, Zhenzhen Xing, Jean-Paul Janssens, Di Chai, Weiming Liu, Yuxia Wang, Yali Ma, Yaqi Tong, Yanfei Guo

**Affiliations:** 1 Department of Respiratory and Critical Care Medicine, Beijing Hospital, National Center of Gerontology, Institute of Geriatric Medicine, Chinese Academy of Medical Sciences, Beijing, China; 2 Departments of Pulmonary and Critical Care Medicine, Peking Union Medical College Hospital, Chinese Academy of Medical Sciences & Peking Union Medical College, Beijing, China; 3 Division of Pulmonary Diseases, Department of Medicine, Geneva University Hospitals, Geneva, Switzerland; 4 Department of Intensive Care Medicine, Beijing Boai Hospital, Beijing, China; 5 Rehabilitation Research Center, Beijing, China

**Keywords:** LLN, Diagnosis, COPD, Spirometry, Lung function

## Abstract

**Background:**

There is no general agreement on the preferential use of a fixed ratio (FR) of forced expiratory volume in 1 s (FEV_1_)/forced vital capacity (FVC) < 0.7 vs. the lower limit of normal (LLN) of FEV_1_/FVC to define airflow obstruction. Determining the impact of these different cut-off levels in people living at high altitudes has not been studied. We assessed the prevalence of airflow obstruction and its clinical characteristics in residents living at high altitude using a fixed ratio and the LLN of FEV_1_/FVC according to Global Lung Initiative 2012 (GLI) reference values.

**Methods:**

Using a multistage stratified sampling method, 3702 participants (aged ≥ 15 years) living at an altitude of 3000–4700 m in Tibet were included.

**Results:**

11.4% and 7.7% of participants had airflow obstruction according to GLI-LLN and a fixed FEV_1_/FVC cut-off value, respectively. The participants in the FR−/LLN+ group were younger, predominantly female, more frequently exposed to household air pollution, and had a higher proportion of chronic obstructive pulmonary disease assessment test scores ≥ 10 than those in the FR−/LLN− group. They also had a significantly lower FEV_1_ and a higher frequency of small airway dysfunction. Compared with the participants of the FR+/LLN+ group, those in the FR−/LLN+ group showed no significant difference in the risk factors for airflow obstruction and respiratory symptoms, but had a lower prevalence of small airway dysfunction.

**Conclusions:**

Defining airflow obstruction according to LLN, instead of using an FR, identified younger individuals with more frequent clinical symptoms of airflow obstruction and small airway dysfunction.

## Introduction

Chronic obstructive pulmonary disease (COPD) is a common preventable but progressive disease that causes heavy economic and social burden. The China Pulmonary Health study (CPH) reported an overall COPD prevalence of 8.6% in the Chinese population aged ≥ 20 years [1]. Spirometry plays a pivotal role in the diagnosis and follow-up of respiratory diseases. The diagnostic criteria for COPD, which are characterized by irreversible airflow obstruction (AO), have been a subject of debate in recent years. The Global Initiative for Chronic Obstructive Lung Disease (GOLD) recommends using a post-bronchodilator (post-) forced expiratory volume in 1 s (FEV_1_)/forced vital capacity (FVC) ratio value < 0.7 for the diagnosis of AO [2]. However, using a fixed ratio (FR) does not consider that FEV_1_/FVC varies with age, height, sex, and ethnicity [2–4]. The GOLD guidelines also point out that using a fixed ratio as a diagnostic criterion may result in over-diagnosis of AO in older individuals and underdiagnosis among younger individuals [2]. The lower limit of normal (LLN) is the fifth percentile of the age-, sex-, and race-specific FEV_1_/FVC ratios. The American Thoracic Society (ATS) and the European Respiratory Society (ERS) favor the use of LLN to diagnose AO [5]. In 2012, the Global Lung Function Initiative (GLI) published the first multiethnic lung function equation for individuals aged 3–95 years providing predicted values and LLN [4]. The GLI reference values are based on 74,000 healthy non-smokers from several respiratory societies.

Worldwide, approximately 400 million people live at high altitudes (> 1500 m above sea level) [6]. People at high altitudes have to endure lower partial pressures of O_2_, cold ambient temperatures, lower ambient humidity, and limited access to medical resources. Compared to individuals living in low-altitude areas, high-altitude residents have undergone physiological and/or genetic changes in their respiratory and circulatory systems [7, 8]. Owing to the specific characteristics of these populations, using an FR may not be appropriate for people at high altitudes.

Although FR < 0.7 is considered the “gold standard” for diagnosing COPD in many guidelines, it may not be applicable in populations at high altitude due to the specific respiratory systems influenced by the environment and genetics. To the best of our knowledge, no studies have compared the FR and LLN criteria in individuals living above 3000 m using the FR and LLN criteria. In the present study, we aimed to compare LLN to FR for the diagnosis of AO in permanent residents of Tibet who were living at high altitude and older than 15 years of age.

## Methods

### Study design and participants

Detailed information regarding the study design and participants has been published elsewhere [9]. Using a multistage stratified sampling procedure, we selected a representative sample of individuals living in Tibet between June 2015 and August 2016. First, the following sites were selected: Lhasa Chengguan District (altitude: 3650 m above sea level), Shigatse City (3900 m), Linzhi County (3000 m), Anduo County (4700 m), Xietongmen County (4100 m), and Duilongdeqing County (4500 m). Second, we selected two streets or townships from each urban or rural area using a simple random sampling method. Third, three communities or village communities were selected from each street or township, using the same method. Finally, using the same method again, individuals from each sex/age stratum were selected from communities or villages.

The exclusion criteria were as follows: tuberculosis treatment during the study period, blood pressure > 180/120 mmHg or heart rate > 120 beats/min, myocardial infarction or cerebrovascular accident within 3 months prior to inclusion, pregnancy, or any condition that would impede the use of spirometry (such as recent thoracic, abdominal, or eye surgery, or retinal detachment).

This study protocol was approved by the Institutional Review Board and ethics committee of Beijing Hospital (2013BJYYEC-042C-01). Before data collection, each individuals received detailed information about the study, and provided informed consent.

### Questionnaires and spirometry

Questionnaire interviews, including potential risk factors for airway obstruction (smoking history, history of tuberculosis, household air pollution (HAP), and exposure to dust or chemicals in the workplace), respiratory symptoms (frequent cough, sputum, recurrent wheezing, and dyspnea in daily life), and the COPD assessment test (CAT), were performed by trained and certified staff. The definitions of these terms have been previously reported [10].

Ambient temperature and humidity were recorded during all spirometric measurements. The spirometer was calibrated daily using a 3-L syringe to include the error correction of syringe volume ≤ ± 3%. Spirometric data were collected using a MasterScreen™ Pneumo PC spirometer (CareFusion, Yorba Linda, CA, USA). Participants were tested twice, before and after inhalation of a bronchodilator (400 µg of salbutamol through a 500 mL spacer). Forced expiratory maneuvers were performed at least three times but no more than eight times. FVC and FEV_1_ were considered to have acceptable repeatability when the difference between the largest and next largest values did not exceed 150 mL. If the three tests failed to reach the standard, the test was repeated. All spirometric measurements and quality control were performed according to ATS/ERS standards and classified into A–F quality levels [11]: a referred to a variability of FVC of 100 mL or less; B, a variability of 100–150 mL; and C, a variability of 150–200 mL. Participants with spirograms of levels A–C were eligible for inclusion in the study.

According to previous recommendations [12, 13] maximal mid-expiratory flow (MMEF), forced expiratory flow (FEF) at 50% of vital capacity, and FEF at 75% of lung function were used to assess small airway obstruction. We defined small airway obstruction as having at least two of these three indicators below 65% of the post-bronchodilator predicted values [14].

### Diagnosis of AO

AO was defined using two post-bronchodilator spirometric criteria: (1) an FR criterion: FEV_1_/FVC < 0.7 and (2) GLI 2012 criterion: FEV_1_/FVC < LLN.

We then classified all participants into four groups based on the FR and LLN criteria:

1. FR−/LLN− (no AO according to both diagnostic criteria)
2. FR−/LLN+ (AO diagnosed by LLN but not by FR)
3. FR+/LLN− (AO diagnosed by FR but not by LLN)
4. FR+/LLN+ (AO diagnosed by both diagnostic criteria).

### Statistical analysis

The characteristics of the FR−/LLN+ group were compared with those of the FR−/LLN− and FR+/LLN+ groups. We analyzed the differences between subgroups after adjust age, gender, BMI, symptoms and risk factors using a multivariate logistic regression model. We included most of the risk factors associated with COPD (age, gender, BMI, history of tuberculosis, household air pollution, exposure in the workplace, and smoking history) as well as respiratory symptoms (one of frequent cough, sputum, recurrent wheezing, and dyspnea in daily life) and CAT score. Spirometric data were analyzed using analysis of covariance with sex and subgroups as fixed variables, and age and body mass index (BMI) as covariates. We examined the differences in the prevalence of small airway obstruction in the subgroups using the Chi-square test. Differences were considered statistically significant at two-sided *P*-values < 0.05. Statistical analyses were performed using SPSS 25.0 for Windows (SPSS Corporation, Chicago, IL, USA).

## Results

**Fig. 1:**
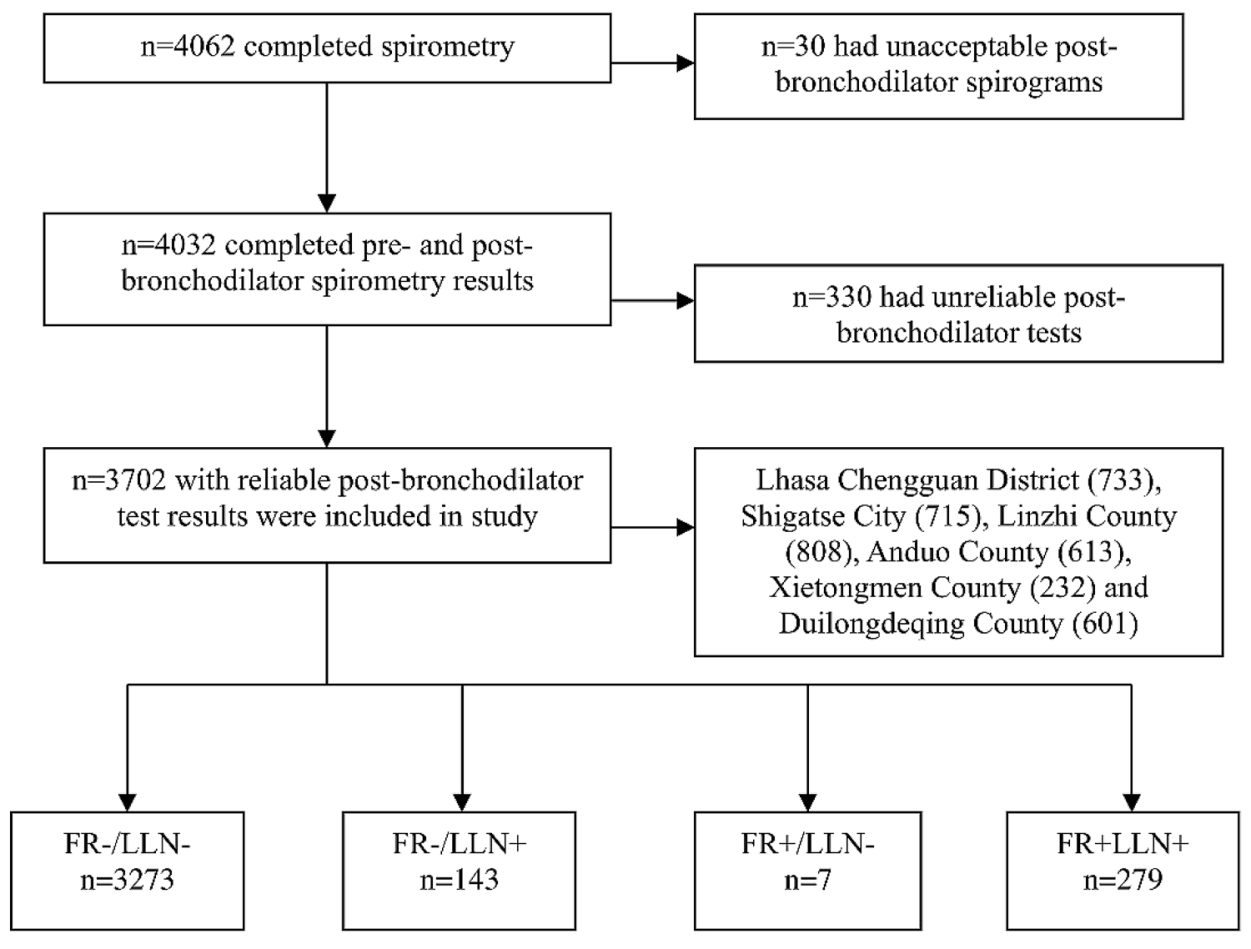
Flowchart of study

After random sampling and excluding individuals with unreliable post-bronchodilator tests, 3702 people aged ≥ 15 years (mean age 39.0 ± 13.9 years, 49.4% male) were included in the final analysis (Fig. 1). The demographic characteristics are summarized in Table 1. Individuals living at high altitudes had a high prevalence of previous tuberculosis history (5.2%). Compared to males, females were more frequently exposed to HAP (67.1 vs. 54.3%) but had a less frequent history of smoking (6.5 vs. 44.1%).

**Table 1:** Demographic characteristics of Tibetan residents by sex: anthropometric data, education, living altitude, risk factors for COPD, smoking status, respiratory symptoms, and CAT score

	Total	Male	Female
*N*	3702 (100)	1828 (49.4)	1874 (50.6)
Age—years	39.0 (13.9)	37.3 (13.7)	40.6 (13.9)
BMI—kg/m^2^	24.1 (4.0)	23.9 (3.7)	24.3 (4.3)
Risk factors for COPD			
Smoking history—no. (%)	928 (25.1)	806 (44.1)	122 (6.5)
History of tuberculosis—no. (%)	193 (5.2)	88 (4.8)	105 (5.6)
Household air pollution^a^—no. (%)	2249 (60.8)	992 (54.3)	1257 (67.1)
Exposure to dust or chemicals at workplace^a^—no. (%)	122 (3.3)	60 (3.3)	62 (3.3)
Respiratory symptom			
Frequent cough^a^—no. (%)	481 (13.0)	250 (13.7)	231 (12.3)
Sputum^a^—no. (%)	466 (12.6)	284 (15.5)	182 (9.7)
Recurrent wheezing^a^—no. (%)	152 (4.1)	57 (3.1)	95 (5.1)
Dyspnea in daily life^a^—no. (%)	1047 (28.3)	457 (25.0)	590 (31.5)
At least one of the above^a^—no. (%)	1471 (39.7)	713 (39.0)	758 (40.4)
CAT score^a^			
< 10—no. (%)	696 (18.8)	349 (19.1)	347 (18.5)
≥ 10—no. (%)	2908 (78.6)	1435 (78.5)	1473 (78.6)
Spirometry—% of predicted			
Post-FEV_1_	106.5 (24.8)	103.1 (22.4)	109.8 (26.5)
Post-FVC	105.5 (26.3)	101.8 (21.9)	109.2 (29.6)
Post-FEV_1_/FVC^b^	84.5 (9.9)	84.8 (10.2)	84.3 (9.6)
Post-MMEF^a^	82.5 (34.5)	85.5 (35.1)	79.5 (33.8)
Post-FEF 50%^a^	98.7 (34.5)	104.0 (35.4)	93.5 (32.9)
Post-FEF 75%^a^	105.9 (53.1)	110.3 (53.6)	101.5 (52.3)

Data are presented as the mean ± standard deviation or number %

BMI, body mass index; CAT, COPD assessment test; COPD, chronic obstructive pulmonary disease

^a^Data missing for household air pollution (*n* = 15), exposure to dust or chemicals in the workplace (*n* = 36), cough (*n* = 82), sputum (*n* = 85), wheezing (*n* = 86), dyspnea (*n* = 81), at least one respiratory symptom (*n* = 80), the CAT score (*n* = 80), MMEF (*n* = 123), FEF 50% (*n* = 7), and FEF 75% (*n* = 7)

^b^Absolute value

**Fig. 2:**
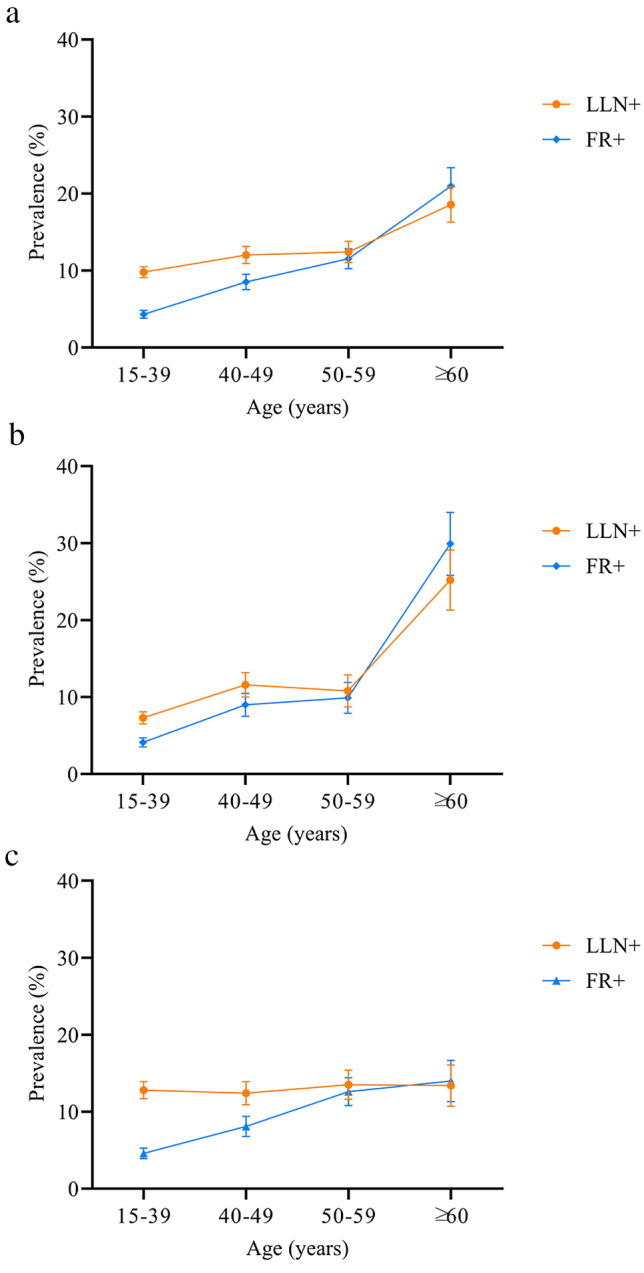
Prevalence of COPD using LLN or FR criteria by age group for **a** all subjects, **b** male and **c** female

**Fig. 3:**
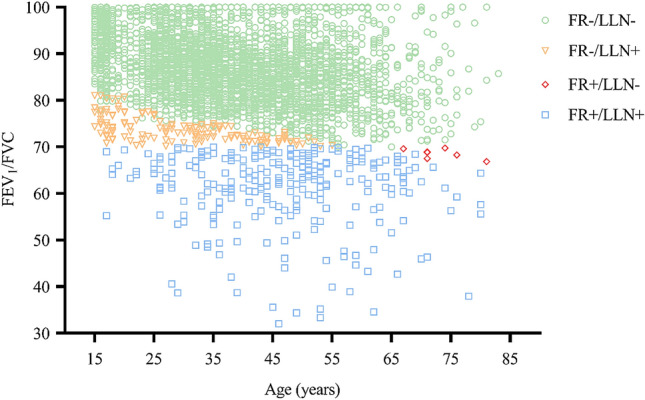
Age against post-bronchodilator FEV_1_/FVC in the study population

The FR−/LLN− group comprised 3,273 (88.4%) individuals, the FR−/LLN+ group: 143 (3.9%), the FR+/LLN− group: 7 (0.2%), and the FR+/LLN+ group: 279 (7.5%) (Table 2). For all individuals, the prevalence of AO was 7.7% (95% CI 6.9–8.6%) based on FR: 7.7% (95% CI 6.4–8.9%) in males and 7.8% (95% CI 6.4–9.0%) in females and 11.4% (95% CI 10.4–12.4%) based on LLN: 9.9% (95% CI 8.5–11.3%) in males and 12.9% (95% CI 11.3–14.4%) in females. The prevalence of AO according to both the diagnostic criteria by age group is shown in Fig. 2. Among young and middle-aged individuals, the prevalence of AO according to LLN was significantly higher than that according to the FR (Figs. 2 and 3). Conversely, in older individuals, the prevalence of AO based on the FR was higher than that based on LLN.

**Table 2:** Characteristics distribution of the individuals in each subgroup according to the two criteria

	FR+/LLN− group (*n* = 7, 0.2%)	FR−/LLN+ group (*n* = 143, 3.9%)	FR−/LLN− group (*n* = 3273, 88.4%)	FR+/LLN+ group (n = 279, 7.5%)	*P* value^a^	
					FR−/LLN+ group vs. FR−/LLN− group	FR−/LLN+ group vs. FR+/LLN+ group
Age—years	73.0 (4.5)	30.1 (11.0)	38.6 (13.6)	47.0 (13.5)	< 0.001	< 0.001
BMI—kg/m^2^	25.5 (3.0)	23.0 (3.7)	24.1 (4.1)	23.9 (3.6)	0.895	0.041
Male—*N*	6 (85.7)	47 (32.9)	1641 (50.1)	134 (48.0)	< 0.001	0.001
History of tuberculosis	1 (14.3)	10 (7.0)	155 (4.7)	27 (9.7)	0.064	0.618
Household air pollution^b^	3 (42.9)	108 (75.5)	1924 (58.8)	214 (76.7)	0.002	0.974
Exposure in the workplace^b^	1 (14.3)	3 (2.1)	105 (3.2)	13 (4.7)	0.567	0.602
Smoking history	4 (57.1)	24 (16.8)	820 (25.1)	80 (28.7)	0.480	0.175
Symptoms^b^	5 (71.4)	55 (38.5)	1251 (38.2)	160 (57.3)	0.356	0.282
CAT score^b^ ≥ 10	5 (71.4)	119 (83.2)	2538 (77.5)	246 (88.2)	0.011	0.488

Data are presented as the mean ± standard deviation or number %

BMI, body mass index; CAT, COPD assessment test; COPD, chronic obstructive pulmonary disease

^a^Analyses of differences using logistic regression. All models were adjusted for age, BMI, sex, risk factors for COPD, smoking status, CAT score, and respiratory symptoms

^b^Data missing for household air pollution (*n* = 15), exposure to dust or chemicals in the workplace (*n* = 36), respiratory symptoms (*n* = 80), and CAT scores (*n* = 80)

According to the different criteria for AO, individuals were assigned to one of the four subgroups. Figure 3 shows the distribution of FEV_1_/FVC according to age into four subgroups.

The clinical characteristics of the subgroups are shown in Table 2. The FR+/LLN group was not compared with other groups because it included only seven participants.

### FR−/LLN+ vs. FR−/LLN− (no AO)

Individuals in the FR−/LLN+ group were younger, largely female, and more exposed to HAP than those in the without AO group. The proportion of individuals with CAT scores ≥ 10 was significantly higher in the FR−/LLN+ group. After adjusting for age, BMI, and sex, participants in the FR−/LLN+ group had a lower post-bronchodilator (post-) percentage of predicted values for FEV_1_, MMEF, FEF 50%, and FEF 75% than that of those in the FR−/LLN− group (Table 3).

**Table 3:** Lung function of the individuals in each subgroup according to the two criteria

	FR−/LLN− group (*n* = 3273)	FR−/LLN+ group (*n* = 143)	FR+/LLN− group (*n* = 7)	FR+/LLN+ group (*n* = 279)	*P* value^a^	
					FR−/LLN+ group vs. FR−/LLN− group	FR−/LLN+ group vs. FR+/LLN+ group
FEV_1_—L	3.3 (0.9)	2.8 (0.7)	2.5 (0.9)	2.4 (0.8)	< 0.001	0.004
FEV_1_—% predicted	108.8 (23.9)	92.7 (19.6)	98.7 (21.1)	86.1 (26.0)	< 0.001	< 0.001
FVC—L	3.8 (1.0)	3.8 (1.0)	3.6 (1.2)	3.9 (1.1)	0.188	0.013
FVC—% predicted	104.6 (25.6)	111.1 (29.8)	103.5 (29.0)	113.8 (30.4)	0.023	0.105
FEV_1_/FVC—%	87.0 (6.8)	73.6 (2.6)	68.5 (1.1)	61.2 (8.2)	< 0.001	< 0.001
MMEF^b^—% predicted	87.6 (32.9)	54.0 (14.5)	40.9 (13.9)	37.8 (16.1)	< 0.001	0.004
FEF 50%^b^—% predicted	104.9 (31.2)	62.4 (16.1)	58.4 (17.7)	45.6 (17.3)	< 0.001	0.001
FEF 75%^b^—% predicted	112.7 (51.5)	58.5 (24.8)	46.1 (18.6)	50.9 (34.9)	< 0.001	0.051

Data are presented as the mean ± standard deviation

BMI, body mass index; FEV1, forced expiratory volume in 1 s; FVC, forced vital capacity; MMEF, maximal mid-expiratory flow; FEF 50%, forced expiratory flow at 50% of vital capacity; FEF 75%, forced expiratory flow at 75% of vital capacity

^a^Spirometric data: *P*-values reported for analysis of covariance with sex and subgroups as fixed variables, and age and BMI as covariates

^b^Data missing for MMEF (*n* = 123), FEF 50% (*n* = 7), and FEF 75% (*n* = 7)

### FR−/LLN+ vs. FR+/LLN+

Individuals in the FR−/LLN+ group were younger, largely female, and had a lower BMI than those in the FR+/LLN+ group (Table 2). There was no difference in the proportion of individuals with risk factors for AO, respiratory symptoms, or CAT scores ≥ 10 between the groups. The significant differences in Pulmonary Function Test were predicted values for post-FEV_1_, post-MMEF, and post-FEF50%, which were all higher in the FR−/LLN+ group (Table 3).

**Fig. 4:**
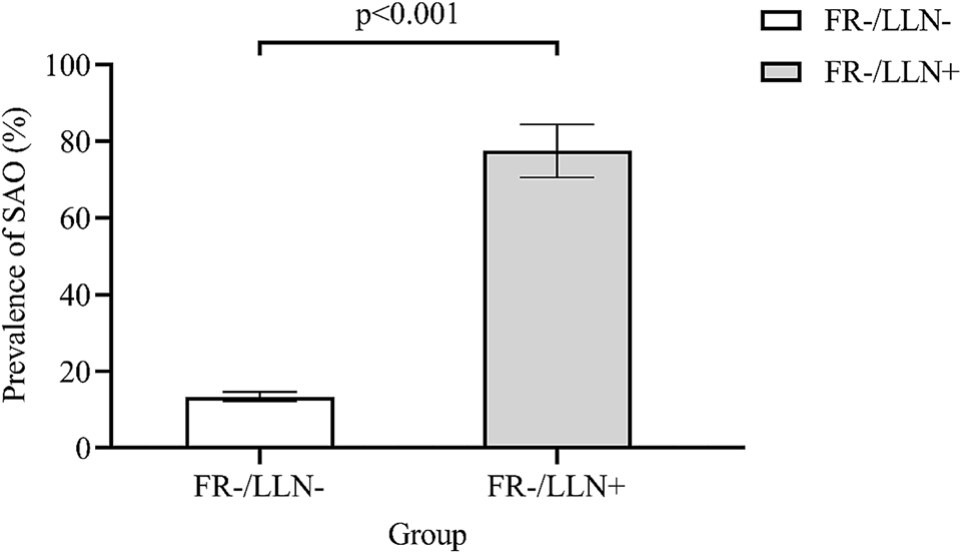
Prevalence of small airway obstruction in each group. Values are shown as mean ± 95% confidence interval. SAO, small airway obstruction. It was defined as at least two of maximal mid-expiratory flow, forced expiratory flow (FEF) 50% and FEF 75% having below 65% of the predicted values. Data missing for FR−/LLN− group (*n* = 31), and FR−/LLN+ group (*n* = 1)

Figure 4 shows that the FR−/LLN+ group had a higher prevalence of small airway obstruction than that of the FR−/LLN− group (77.5 vs. 13.4%, *P* < 0.001).

## Discussion

In high altitude dwelling subjects (> 3000 m) living in Tibet and aged over 15 years, this large-sample epidemiological study evaluated the prevalence of COPD using two criteria: the LLN based on GLI 2012 reference equations and a fixed FEV_1_/FVC ratio as proposed by GOLD, based on post-bronchodilator lung function values. Among participants, 92% performed spirograms fulfilling ATS/ERS quality criteria. The present data show that using the LLN as a criterion for defining AO resulted in a higher proportion of subjects classified as having AO compared to using a fixed FEV_1_/FVC ratio criterion (LLN: 11.4 vs. FR: 7.7%).

When compared to individuals in the FR−/LLN− group, those in the FR−/LLN+ group (i.e., AO identified only by LLN) were younger, predominantly female, and had a worse post-FEV_1_ predicted and FEV_1_/FVC ratio.

Tibet has an average altitude of over 4000 m. Its permanent population is about 365 million, of which the Tibetan population represents 314 million (86%) [15]. In active smokers, living at high altitudes is associated with an accelerated decline in lung function [16]. Inhabitants living at high altitude on a long-term basis have undergone genetic changes, which affect regulation of breathing [8]. Genome-wide scans of Tibetans have identified candidate genes involved in adapting to high-altitude hypoxia [7]. To determine the correlation between altitude and COPD prevalence, Horner et al. analyzed lung function of 30,874 individuals from 44 sites worldwide. They found that both FVC and FEV_1_ were significantly lower at higher altitude and that the FEV_1_/FVC ratio decreased because FEV_1_ decreased proportionally more than FVC, thus impacting on the ability of a FR to identify the population with AO at a high altitude [17]. Potential mechanisms behind these changes in lung function include harsh living environment, poverty, low birth weight, exposure to HAP [18–20]; adapting to chronic hypoxia and the related increase in ventilation [17]. However, to the best of our knowledge, no study has focused on comparing spirometry criteria for AO at high altitudes.

Prevalence of AO by LLN was higher than by FR for young and middle-aged individuals, while the opposite was true in older individuals. The potential for underdiagnosis of AO using the FR criterion in young and middle-aged individuals has been previously demonstrated [21–23]. Mild AO in young individuals may be related to early stage COPD. Young adults with early COPD report chronic respiratory symptoms and severe lung function impairment more often and show a high incidence of acute respiratory hospitalizations and early death [24]. We considered that individuals in the FR−/LLN+ group differed from those of the normal group in terms of airway impairment and suffer from significant airway disease. These findings were consistent with previous studies in China [25]. Early diagnosis of this population may have a positive impact on preventive measures, optimal medical management, and may help slow disease progression.

It is well recognized that the FR criterion will not only miss the diagnosis of AO in the younger population, but also lead to over-diagnosis it in older individuals [23, 25, 26]. Çolak et al. studied 95,288 individuals aged 20–100 years and found that > 99% of the individuals in the FR+/LLN− group were aged > 50 years [23]. After a 6-year follow-up, individuals with potentially over-diagnosed airflow limitation (FR+/LLN− group) had all-cause mortality similar to individuals without airflow limitation. Thus, age must be taken into account when using the FR criterion for diagnosing AO.

Subjects with AO according LLN have a poor prognosis [23, 26–28]. A follow-up study of 181,300 person-years showed that subjects classified as FR−/LLN+ had the highest risk of death than normal population (HR = 4.04) [27]. It seems that, compared to the fixed ratio, LLN correlates better with the prognosis of COPD.

The small airway indicators (MMEF, FEF50%, and FEF75%) values of the FR−/LLN+ group were significantly lower in the FR−/LLN− group than in the FR−/LLN− group. Small airway impairment is regarded as a precursor to COPD. Indeed, McDonough et al. emphasized that narrowing and loss of small airways preceded emphysema in COPD [29]. Xiao et al. advocated that measuring small airway function may allow identifying people at early stages of COPD or at high risk of developing COPD [13].

Compared with that of the FR−/LLN− group, both FR−/LLN+ and FR+/LLN+ groups had been more exposed to HAP, which may have contributed to their lung function impairment: 2.6 billion people (approximately 40% of the world’s population) cook with polluting biomass fuels [30]. Exposure to HAP is one of the risk factors for COPD [20, 31], causing an estimated 3.8 million deaths every year. More than 90% of air pollution-related deaths occur in low-income and middle-income countries, mainly Asia and Africa [30]. The proportion of individuals exposed to HAP in our study was high (60.8%). People at high altitudes require more heating owing to extreme climatic conditions while limiting ventilation of their living environment to preserve warmth [20]. The proportion of female individuals exposed to HAP owing to cooking and heating was higher than that of the male population (67.1% vs. 54.3%). HAP is associated with adverse respiratory diseases in women [32]: women exposed to high levels of biomass fuels are more than twice as likely to suffer from COPD as those exposed to cleaner fuels and technologies [33].

FR−/LLN+ individuals also have a high probability of reporting respiratory symptoms [23, 25, 34]. The CAT score is widely used to assess COPD symptoms. In the present study, more FR−/LLN+ subjects had a CAT ≥ 10 when compared to FR−/LLN− group.

There are some strengths about our study. First, this is the first study comparing the FR and LLN for diagnosis of airflow obstruction at high altitude over 3000 m. Appropriate cut-off points for the FEV_1_/FVC ratio are crucial for proper COPD diagnosis, treatment, and apportionment of scarce resources such as supplemental oxygen for people in high altitude. Second, our diagnosis of AO is based on post-bronchodilator spirometry to avoid reversibility according to GOLD guidelines. Third, detailed data on risk factors, and spirometry were collected, and included in in multivariate analyses.

Our study has a few limitations. First, because this was a cross-sectional study, we could not make definitive conclusions as to comparison of mortality and hospitalization rates between both diagnostic criteria. Second, the missing data may have biased the results, although their amount was small. Third, the small size of the FR+/LLN− group limited the possibility of statistical analysis, was the case in similar studies [25, 35].

By collecting, analyzing and synthesizing population-level data, our studies can provide insight into the distribution of AO and identify the particular population at risk for COPD in Tibet. We found FR−/LLN+ group showed higher exposure rate of HAP, which is the important risk factors for COPD. As cooler temperatures and lower incomes, residents at high altitudes preference for increased use of solid fuel-based heating. Addition, adequate ventilation and air purification facilities are not available locally. It provided useful information for government to consider stringent solid fuels control measures and improve poor living conditions. Another remarkable result was FR−/LLN+ group showed more negative values of lung function and higher prevalence of SAO than FR−/LLN− group. Respiratory symptoms of people in high altitude may affected by air condition, so spirometry is particularly important for diagnosis and screening of airway obstruction. And most importantly, people in the FR−LLN+ group were young. Lung function may deteriorate with age. Even if they cannot diagnose COPD based on the guide of GOLD, local physician should recommend them to follow up and monitor changes of lung function.

## Conclusions

Our study showed that the prevalence of AO among people at high altitudes differs greatly when different criteria are used for defining AO. Among young and middle-aged individuals, the prevalence of AO by LLN was significantly higher than that by FR. Conversely, in older individuals, the prevalence of AO according to the FR was higher. Defining AO according to LLN instead of FR in a younger ages identified more female participants with clinical conditions that were compatible with AO, such as exposure to HAP and a CAT score ≥ 10, who require a longitudinal medical follow-up.

## Data Availability

The datasets used or analyzed during the current study are available from the corresponding author on reasonable request.

## Abbreviations

FR: Fixed ratio
FEV1: Forced expiratory volume in 1 s
FVC: Forced vital capacity
LLN: Lower limit of normal
GLI: Global Lung Initiative 2012
COPD: Chronic obstructive pulmonary disease
CPH: China Pulmonary Health study
AO: Airflow obstruction
GOLD: Global Initiative for Chronic Obstructive Lung Disease
post-: Post-bronchodilator
ATS: American Thoracic Society
ERS: European Respiratory Society
HAP: Household air pollution
CAT: Chronic obstructive pulmonary disease assessment test
MMEF: Maximal mid-expiratory flow
FEF: Forced expiratory flow
BMI: Body mass index
